# Supplementary material for: Breaking with traditions: Who are the women with attitudes, norms and behaviors that support ending female genital mutilation in Burkina Faso?
Source: PLOS Glob Public Health. 2025 Dec 12;5(12):e0005621. doi: 10.1371/journal.pgph.0005621 (PMC12700381; doi:10.1371/journal.pgph.0005621)
Supplement: S1 Table — (DOCX) [file pgph.0005621.s001.docx]

| **S1 Table. Multinomial logistic regression relative risk ratios (95% CI) of women's readiness for change based on FGM attitudes and behaviors, Burkina Faso PMA, rural (Model 2) and urban (Model 3) women who had FGM** | | | | | | |
| --- | --- | --- | --- | --- | --- | --- |
|  |  | **Model 2 - Rural women who had FGM** | | | | |
|  |  | Reluctant adherent | Reluctant abandoner | Willing abandoner | Contemplator | Reluctant adherent |
|  |  | vs. | vs. | vs. | vs. | vs. |
| Characteristic |  | Willing adherent | Willing adherent | Willing adherent | Willing adherent | Willing abandoner |
| Education level (Ref: No formal education) |  |  |  |  |  |  |
| Primary |  | 1.11 (0.65-1.87) | 0.67 (0.26-1.72) | 1.10 (0.61-1.98) | 0.94 (0.33-2.62) | 1.00 (0.82-1.24) |
| Secondary or higher |  | 1.25 (0.98-1.60)+ | 0.66 (0.20-2.19) | 2.10 (1.58-2.79)*** | 0.92 (0.39-2.15) | 0.60 (0.48-0.75)*** |
| Age group (Ref: 35+) |  |  |  |  |  |  |
| 15-24 |  | 0.25 (0.09-0.69)* | 0.21 (0.07-0.58)*** | 0.21 (0.09-0.52)** | 0.19 (0.03-1.01)+ | 1.19 (0.22-6.52) |
| 25-34 |  | 0.59 (0.35-0.98)* | 0.66 (0.21-2.10) | 0.44 (0.30-0.65)*** | 0.91 (0.59-1.42) | 1.34 (0.70-2.56) |
| Residence (Ref: Rural) |  |  |  |  |  |  |
| Urban |  | na | na | na | na | na |
| Religion (Ref: Christian)¥ |  |  |  |  |  |  |
| Muslim |  | 0.84 (0.46-1.52) | 0.80 (0.26-2.45) | 0.31 (0.20-0.49)*** | 1.29 (0.69-2.40) | 2.68 (1.22-5.89)* |
| Traditional religion |  | 1.12 (0.24-5.17) | 2.66 (0.31-22.76) | 1.17 (0.66-2.07) | 0.76 (0.33-1.71) | 0.96 (0.33-2.82) |
| Parity (Ref: None) |  |  |  |  |  |  |
| 1-2 |  | 0.87 (0.39-1.98) | 0.66 (0.20-2.13) | 1.40 (0.55-3.58) | 0.30 (0.12-0.72)* | 0.62 (0.44-0.88)* |
| 3-4 |  | 0.75 (0.41-1.34) | 0.33 (0.10-1.09)+ | 1.18 (0.37-3..74) | 0.31 (0.06-1.49) | 0.63 (0.28-1.41) |
| 5+ |  | 1.22 (0.43-3.44) | 0.46 (0.10-2.04) | 1.24 (0.29-5.31) | 0.41 (0.06-2.63) | 0.99 (0.34-2.87) |
| Living arrangement |  |  |  |  |  |  |
| Single/widowed/divorced (ref) | | |  |  |  |  |
| Married/living together |  | 0.85 (0.61-1.19) | 4.01 (1.34-11.99)* | 0.85 (0.60-1.22) | 1.39 (0.73-2.64) | 1.00 (0.74-1.35) |
| Men in community make household decisions | | |  |  |  |  |
| Disagree (ref) |  |  |  |  |  |  |
| Agree |  | 0.99 (0.62-1.56) | 1.77 (0.43-7.31) | 1.07 (0.80-1.43) | 0.75 (0.27-2.07) | 0.92 (0.56-1.51) |
| Worked in the last 7 days (Ref: No) | | |  |  |  |  |
| Yes worked |  | 1.06 (0.55-2.04) | 0.54 (0.28-1.03)+ | 0.89 (0.59-1.35) | 0.90 (0.53-1.53) | 1.18 (0.71-1.96) |
| Number of observations (unweighted) |  | n=1,579 | | | | |
| Note outcome is readiness category (based on attitude on whether FGM should be abandoned or continued and whether she performed or would perform FGM on daughter); +p ≤ 0.10; *p ≤ 0.05; **p ≤ 0.01; ***p ≤ 0.001; ¥ Note religion is based on head of household's reported religion | | | | | | |
| **S1 Table continued.** | | | | | | |
|  |  | **Model 3 - urban women who had FGM** | | | | |
|  |  | Reluctant adherent | Reluctant abandoner | Willing abandoner | Contemplator | Reluctant adherent |
|  |  | vs. | vs. | vs. | vs. | vs. |
| Characteristic |  | Willing adherent | Willing adherent | Willing adherent | Willing adherent | Willing abandoner |
| Education level (Ref: No formal education) |  |  |  |  |  |  |
| Primary |  | 1.05 (0.64-1.70) | 2.01 (1.07-3.74)* | 1.14 (0.92-1.42) | 0.86 (0.57-1.30) | 0.92 (0.57-1.46) |
| Secondary or higher |  | 1.73 (1.01-2.96)* | 2.16 (1.09-4.29)* | 3.86 (2.90-5.14)*** | 1.12 (0.54-2.32) | 0.45 (0.28-0.73)** |
| Age group (Ref: 35+) |  |  |  |  |  |  |
| 15-24 |  | 0.47 (0.16-1.37) | 0.63 (0.19-2.07) | 0.17 (0.08-0.34)*** | 0.24 (0.12-0.50)*** | 2.81 (1.16-6.81)* |
| 25-34 |  | 0.50 (0.28-0.91)* | 1.06 (0.44-2.57) | 0.54 (0.36-0.82)** | 0.70 (0.43-1.13) | 0.93 (0.56-1.56) |
| Residence (Ref: Rural) |  |  |  |  |  |  |
| Urban |  | na | na | na | na | na |
| Religion (Ref: Christian)¥ |  |  |  |  |  |  |
| Muslim |  | 0.94 (0.60-1.46) | 0.35 (0.24-0.50)*** | 0.30 (0.21-0.43)*** | 0.60 (0.44-0.81)** | 3.15 (1.87-5.32)*** |
| Traditional religion |  | ¥¥ | ¥¥ | ¥¥ | ¥¥ | ¥¥ |
| Parity (Ref: None) |  |  |  |  |  |  |
| 1-2 |  | 1.42 (0.55-3.68) | 2.40 (1.12-5.17)* | 0.81 (0.40-1.63) | 0.86 (0.27-2.73) | 1.75 (0.91-3.37)+ |
| 3-4 |  | 1.91 (0.59-6.25) | 3.94 (1.37-11.34)* | 0.69 (0.29-1.62) | 0.64 (0.25-1.66) | 2.79 (1.22-6.35)* |
| 5+ |  | 2.25 (0.77-6.56) | 3.71 (0.96-14.30)+ | 0.40 (0.16-0.97)* | 0.31 (0.07-1.48) | 5.69 (2.23-14.55)*** |
| Living arrangement |  |  |  |  |  |  |
| Single/widowed/divorced (ref) | | |  |  |  |  |
| Married/living together |  | 0.82 (0.41-1.65) | 0.89 (0.48-1.67) | 1.21 (0.79-1.86) | 1.14 (0.63-2.06) | 0.68 (0.41-1.13) |
| Men in community make household decisions | | |  |  |  |  |
| Disagree (ref) |  |  |  |  |  |  |
| Agree |  | 0.65 (0.50-0.85)** | 2.41 (0.63-9.25) | 0.87 (0.57-1.33) | 1.00 (0.66-1.53) | 0.74 (0.53-1.04)+ |
| Worked in the last 7 days (Ref: No) | | |  |  |  |  |
| Yes worked |  | 1.05 (0.67-1.65) | 0.98 (0.55-1.77) | 1.19 (0.87-1.63) | 1.28 (0.81-2.03) | 0.88 (0.65-1.20) |
| Number of observations (unweighted) |  | n=1,776 | | | | |
| Note outcome is readiness category (based on attitude on whether FGM should be abandoned or continued and whether she performed or would perform FGM on daughter); +p ≤ 0.10; *p ≤ 0.05; **p ≤ 0.01; ***p ≤ 0.001; ¥ Note religion is based on head of household's reported religion. ¥¥ 28 observations with a traditional religion dropped because no variability in outcome (all report innovative outcome) | | | | | | |
