# Supplementary figures and images for: Breaking with traditions: Who are the women with attitudes, norms and behaviors that support ending female genital mutilation in Burkina Faso?
Source: PLOS Glob Public Health. 2025 Dec 12;5(12):e0005621. doi: 10.1371/journal.pgph.0005621 (PMC12700381; doi:10.1371/journal.pgph.0005621)

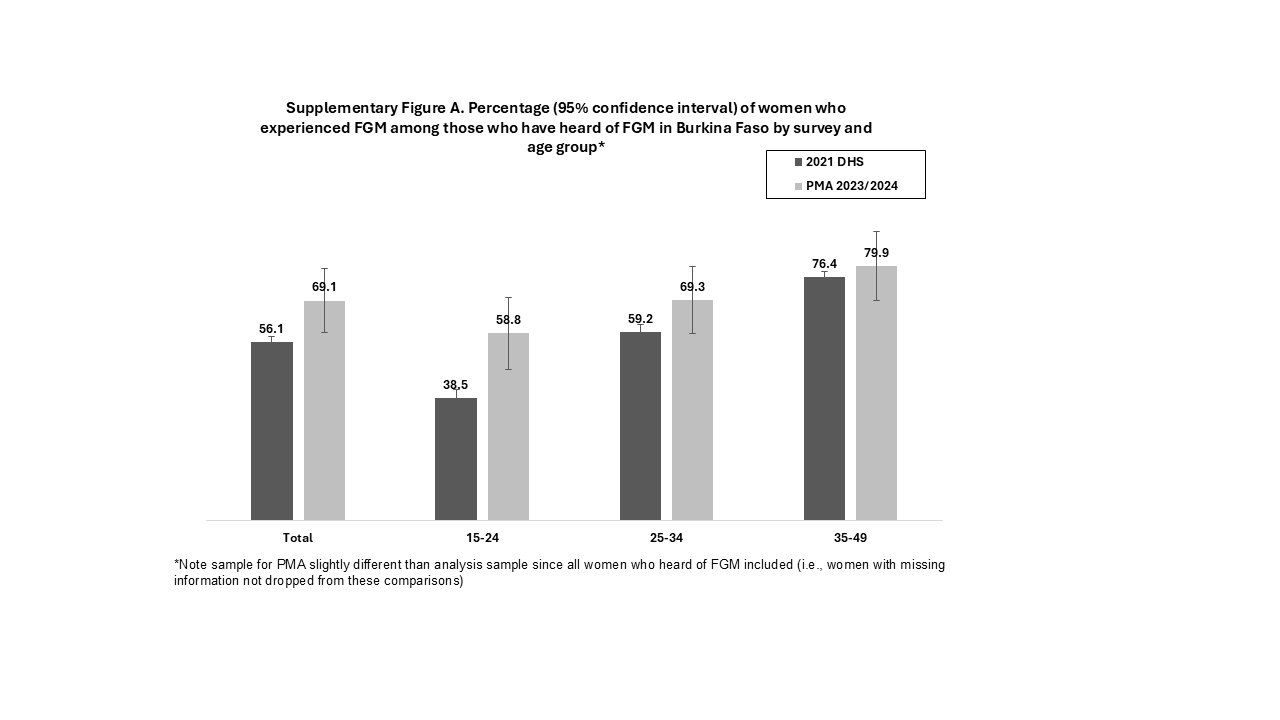

Supplement: S1 Fig — (TIF) [file pgph.0005621.s002.tif]
