# Supplementary material for: Breaking with traditions: Who are the women with attitudes, norms and behaviors that support ending female genital mutilation in Burkina Faso?
Source: PLOS Glob Public Health. 2025 Dec 12;5(12):e0005621. doi: 10.1371/journal.pgph.0005621 (PMC12700381; doi:10.1371/journal.pgph.0005621)
Supplement: Attachment — Submitted filename: Response to reviewers_v2.docx [file pgph.0005621.s004.docx]

Journal Requirements:

1. Please amend your detailed Financial Disclosure statement. This is published with the article. It must therefore be completed in full sentences and contain the exact wording you wish to be published.

a. State the initials, alongside each funding source, of each author to receive each grant.
b. State what role the funders took in the study. If the funders had no role in your study, please state: “The funders had no role in study design, data collection and analysis, decision to publish, or preparation of the manuscript.”
c. If any authors received a salary from any of your funders, please state which authors and which funders.

If you did not receive any funding for this study, please simply state: “The authors received no specific funding for this work.

Response: The financial disclosure statement has been revised.

**Reviewer #1:**

This manuscript examines demographic factors associated with supportive attitudes, norms, and behaviours toward abandoning female genital mutilation (FGM) among women in Burkina Faso, drawing on recently collected, nationally representative PMA data. The research addresses a highly relevant and policy-critical issue and offers potentially actionable insights for program design. The paper is well-written and clearly structured. Nonetheless, the manuscript would benefit from a more Burkina Faso–specific theoretical framing, a deeper conceptualisation and justification of the term and definition of “innovators,” and a more explicit integration of existing evidence—particularly from UNICEF’s 2013 work—on legislation, FGM support, and underreporting in Burkina Faso.

Response: Thank you for these high-level inputs (which are detailed below) which we have taken into consideration in the revised manuscript (and respond to more deeply below).

Major Comments
The introduction currently draws heavily on general or multi-country literature. It should be strengthened by presenting a Burkina Faso–specific theoretical framework. In particular, the seminal UNICEF (2013) study Female Genital Mutilation/Cutting: A statistical overview and exploration of the dynamics of change contains country-level insights on the role of legislation, enforcement, and the underreporting of supportive attitudes due to social desirability bias in survey contexts. These findings could provide essential context for interpreting your results, particularly the unexpectedly high proportion of younger women with pro-continuation views.

Response: We have significantly revised the introduction to incorporate more details and considerations around Burkina Faso as well as the context of measuring FGM in contexts where it is illegal. We have also added a paragraph at the beginning of the methods that provides more details on the Burkina Faso context.

Moreover, the current operational definition of “innovators”—based on a combination of personal attitudes and perceived community norms—feels primarily descriptive. I recommend developing a theoretically grounded definition, explicitly linking it to social norms theory (e.g., injunctive/descriptive norms) and the diffusion of innovations framework. This would provide stronger conceptual underpinnings for your classification and ensure it reflects behavioural science theory rather than the convenience of available data. The definition should be robustly grounded in theory. While the term “innovators” is borrowed from the diffusion of innovations theory (which you could explain in more depth), its use here should be discussed and justified more carefully. After the proposed theoretical reframing, you might consider alternative terminology (e.g., “early adopters of abandonment norms” or “norm-changers”) that better captures the social positioning outside the prevailing norm, without unintended value judgements. The choice of term should follow from the conceptual discussion in the introduction.

Response: These important inputs have been truly appreciated, and the revised introduction frames the relevant behavioral theories, brings in another conceptual approach to assess changes in attitudes and behaviors around FGM (readiness to change) based on the work of Shell-Duncan and Hernlund (2006). We relate our measure based on the attitude and injunctive norms to this readiness to change perspective and include the additional outcome of readiness to change in the paper. We have also adapted the language in the text to identify those with attitudes/norms that are associated with stopping FGM as ‘early adopters of FGM abandoning norms’ as proposed by the review. That said, we still call those who are not performing it on their own daughters “innovators” as they are not practicing a behavior that they themselves experienced.

The finding that younger women with FGM are more likely to support continuation than older women is counterintuitive and warrants deeper theorisation (although it is not entirely new—see Masho and Matthews 2009 for Ethiopia or Ortensi et al. 2025 among migrants in Italy for similar results). Beyond the explanations currently offered, consider, among others:

- Cohort effects linked to the timing of legislation and enforcement in Burkina Faso.

-Potential underreporting of support for continuation among older women due to greater awareness of socially acceptable responses.

- Differences in exposure to abandonment campaigns across age cohorts, including possible effects of COVID-19 on the discontinuation or scaling back of prevention campaigns.

Response: Thank you for these additional references and reflections on this surprising finding. We have incorporated this information into the discussion section.

The difference between PMA and DHS prevalence estimates is noted but could be unpacked further, considering potential methodological drivers such as interviewer training, trust-building via resident enumerators, and question ordering or phrasing effects.

Response: This is now addressed more fully as part of the limitations in the discussion section of the paper.

**Reviewer #2:**

This is an interesting study addressing an important research topic and significant public health issue.
Comments:
1. The title should explicitly include the word “women”, as the term “innovators” could also be interpreted to include men.

Response: The title has been revised accordingly.

2. In lines 78–82, remove the word “Recent” since it refers to the year 2021, which is no longer very recent.

Response: This sentence has been removed/changed and we have kept this in mind in the revising of the introduction.

3. In lines 111–113 (Phase 4 Data of PMA), provide more information about this survey and include a reference if available.

Response: Thank you for this comment, we have added more information to the text with appropriate links to the data source.

4. In line 125, replace the hyperlink with a formal reference.

Response: This change has been made.

5. Clarify whether you obtained approval to access and use the survey data. If so, please state this explicitly.

Response: We have clarified that we have used public release data and have provided the location to request access.

6. In Table 1, it would be more informative to present both numbers and percentages.
7. In Table 1, for the education level variable, use “No formal education” instead of “None.”
8. In Table 1, the percentages for Urban and Rural appear incorrect (should be 40% and 60% instead of 27% and 76%). Please verify these figures. Also, since Urban/Rural are already shown as columns, there is no need to repeat them in the rows.

Responses related to Table 1: Thank you. We have focused the table on the weighted proportions because we felt those were most relevant to understand our sample and findings. However, based on your feedback we have tried to include both weighted and unweighted numbers in the table for those who would like to see them. We have included the unweighted number of observations in the table and in the footnote we have noted that these are unweighted and also provide the overall weighted values for the full sample, the rural sample and the urban sample. As you can see, the weighted sample has more rural than urban residents, as reflected in the total column percentages. We prefer not to include the number of observations in the table cells themselves as the focus is on the distribution by group rather than number within each cell, and we felt that showing both in every cell would reduce the clarity of the table. We have made the requested change to the education classification.

9. In the Materials and Methods section, consider reorganizing it into standard, clearly labeled subsections for better clarity and flow.

Response: Thank you, we have added more subsections in this revision, which we hope will improve clarity.

10. Provide more detail on the statistical analysis in the Materials and Methods section.

Response: Thank you for noticing this gap; this information has been added.

11. The study relies on only 18 references, many of which are organizational reports rather than peer-reviewed academic papers. Given the importance and the well-researched nature of the topic, more literature—especially academic sources—should be incorporated, particularly in the Discussion.

Response: We have now incorporated more theoretical and peer-reviewed references to help frame the study and support the discussion.

12. In the Discussion, expand on the implications of your findings in greater depth, addressing practice, policy, and future research aspects.

Response: We have expanded the discussion section to include more on the practical and policy implications of these findings, based on this and the previous reviewers inputs.
